# Editorial: Adipose Tissue in Obesity and Metabolic Disease

**DOI:** 10.3389/fphys.2022.898861

**Published:** 2022-04-19

**Authors:** Sahar Keshvari, Ryan P. Ceddia, Prashant Rajbhandari, Bhagirath Chaurasia, Simon T. Bond

**Affiliations:** ^1^ Translational Research Institute, Mater Research Institute-The University of Queensland, Brisbane, QLD, Australia; ^2^ Division of Cardiovascular Medicine, Department of Medicine, Vanderbilt University Medical Center, Nashville, TN, United States; ^3^ Diabetes, Obesity, and Metabolism Institute, Icahn School of Medicine Mount Sinai, New York, NY, United States; ^4^ Division of Endocrinology, Department of Internal Medicine, Carver College of Medicine, Fraternal Order of Eagles Diabetes Research Center, University of Iowa, Iowa City, IA, United States; ^5^ Baker Heart and Diabetes Institute, Melbourne, VIC, Australia; ^6^ Baker Department of Cardiometabolic Health, The University of Melbourne, Melbourne, VIC, Australia; ^7^ Central Clinical School, Monash University, Melbourne, VIC, Australia

**Keywords:** adipose, adipose tissue, lipids, brown fat, obesity, metabolism

Adipose tissue is a highly adaptive and critically important metabolic tissue, with intrinsic roles in both health and disease. It is now well established that adipose tissue plays a significant role in a diverse range of physiological processes, including metabolic homeostasis and disease progression. Obesity, a chronic condition defined by excessive adipose tissue expansion, has now become one of the greatest medical challenges of the modern world. Consequently, understanding the mechanisms that underpin adipose tissue in obesity and disease progression is becoming increasingly important, such that novel targets for the treatment of obesity and its complications may be identified. The aim of this Research Topic is to provide an overview of the important physiological roles that adipose tissue plays in both health and disease, by summarising current knowledge and providing novel insights into adipose tissue biology.

Excessive adipose tissue expansion is associated with declines in health and quality of life, however, the type of expansion (hyperplasia or hypertrophy) and the anatomical location of the adipose depot, are now known to be key factors that affect metabolic health. This is nicely summarised in the review by Nunn et al. where they describe the functional differences that occur in hypertrophic adipocytes and how this can contribute to metabolic complications. The authors detail how initiating adipogenesis (hyperplasia) as opposed to hypertrophy is metabolically beneficial and could be targeted therapeutically to treat metabolic disease. The review then evaluates known adipogenic regulators that could potentially be manipulated to induce adipogenesis. The concept of adipose tissue expansion is extended upon in a detailed review by Hilgendorf, which analyses the current literature regarding adipocyte primary cilia and their role in adipose tissue expansion and metabolic disease. The primary cilium is a cellular protrusion found on most mammalian cell types, including adipocytes, where it can regulate pro- and anti-adipogenic signalling pathways. Hilgendorf concludes by postulating that adipocyte primary cilium may function as a signalling hub through ciliary remodelling, enabling the primary cilium to sense and respond to extracellular signals, which can initiate adipogenesis. Garritson and Boudina further elucidate on mechanisms of adipose tissue expansion in a review that describes the effect of exercise on adipose tissue plasticity. It is well known that lifestyle interventions such as diet and exercise can reduce the risk for developing cardiometabolic disorders, but many of the underlying mechanisms remain unknown. Garritson and Boudina summarise the positive metabolic benefits of exercise on adipose tissue and highlight important questions that remain to be addressed.

There are three distinct forms of adipocytes; white (the body’s main depot for storing energy), brown (accrue and burn lipid for energy) and beige (ability to switch between white and brown adipocyte roles). Since the discovery of brown fat in both humans and rodents, there has been significant advances in our understanding of its role in metabolic homeostasis, however, there are still existing knowledge gaps regarding brown fat biology. Although previous research has focused on glucose uptake into brown fat, Wade et al. provide a detailed update on the previously underappreciated role of lipid uptake into activated brown fat. Here, the authors highlight the importance of peripheral lipid storage for non-shivering thermogenesis and present an update on known mechanisms of lipid transport and uptake into brown fat. Researchers have long been looking for ways to artificially stimulate brown fat, such as the identification of novel browning mechanisms, to harness the positive metabolic effects of activated brown fat. Wang et al. original research article demonstrates a previously unknown CLK1-THRAP3-PPARγ axis that plays a role in adipose tissue browning and insulin sensitivity. The authors observe that CLK1 KO mice are resistant to high fat diet with preserved glucose tolerance and insulin sensitivity. They further demonstrate that CLK1 phosphorylates THRAP3 which promotes docking of PPARγ to inhibit PPARγ activity. Moreover, the original research article by Xiong et al. examines the role of the transcription factor E2F1 on mouse white adipose tissue browning and autophagy. In this article the authors demonstrate that global deletion of E2F1 leads to smaller adipocyte cell size and increased mitochondrial content which are associated with reduced expression of genes and proteins related to autophagy. Although activation of brown fat has been shown to be beneficial to metabolic health, the ability to therapeutically target brown adipose tissue has not yet translated to the clinic. Potentially the activation of brown fat in the setting of obesity provides many therapeutic challenges, including a reduction of brown fat in obesity and the difficulty in specifically targeting brown adipose tissue. Bond et al. review on adipose extracellular vesicles summarises the literature regarding the role of adipocyte extracellular vesicles in health and disease, where the authors postulate that extracellular vesicles secreted from activated brown fat may provide a new therapeutic avenue to mimic the metabolic benefits of brown fat activation.

Declines in insulin sensitivity are strongly correlated with obesity and dysfunctional adipose tissue. Original research by Van Meijel et al. investigates proteomic profile alterations in the abdominal subcutaneous adipose tissue from overweight/obese and insulin resistant male subjects caused by mild intermittent hypoxia. Using untargeted liquid chromatography-mass spectrometry the authors identify 123 proteins that are differentially expressed due to mild intermittent hypoxia exposure and find a correlation between adipose tissue insulin sensitivity and changes in TMOD3 expression. Approved treatments to improve insulin sensitivity include the thiazolidinedione class of drugs, which have had mixed success largely due to off target effects, where many of the beneficial and detrimental mechanism remain poorly understood. Original research by Palavicini et al. contributes additional knowledge on how the insulin-sensitiser pioglitazone exerts its cardiometabolic benefits in the setting of type 2 diabetes. Using shotgun lipidomics to measure changes in lipid species from human subcutaneous abdominal adipose tissue and vastus lateralis muscle from obese patients with type 2 diabetes following a chronic 6-month treatment with pioglitazone, the authors determine that the majority of the lipid composition changes occur in the glycerophospholipid pool. Glycerophospholipids enriched for the inflammatory pathway modulator arachidonic acid and its precursor linoleic acid were reduced by pioglitazone highlighting the importance of adipocyte membrane function in immunometabolic health. Adipose lipid metabolism, in particular ether lipids in obesity, is discussed further in a review by Schooneveldt et al *.*, in which they delineate the functional and protective roles of ether lipids in the setting of obesity. The authors discuss how ether lipids have been linked to lipid droplet formation, regulating thermogenesis and mediating browning of white adipose tissue, as well as the therapeutic potential of ether lipid supplementation to alleviate obesity and its associated complications.

The overarching theme of this Research Topic is to better understand adipose tissue biology to uncover new therapeutic targets to treat obesity and metabolic disease. The potential of synthetic oligonucleotide technologies, as a means to target specific genes or proteins, to treat metabolic disease is explored in a review by Keating et al. Here the authors evaluate oligonucleotide-based therapies in the setting of obesity and fatty liver disease, as well as discussing the current limitations and challenges of oligonucleotide-based technologies in pre-clinical studies and their use as therapeutics.

In conclusion, this Research Topic on adipose tissue incorporates a Research Topic of reviews and novel data summarising our current understanding of adipose tissue biology and its role in maintaining metabolic homeostasis. Elucidating the physiological mechanisms of adipose tissue function, in both health and disease, could help to uncover novel therapeutic targets to treat obesity and metabolic disease.

